# Free radicals and their impact on health and antioxidant defenses: a review

**DOI:** 10.1038/s41420-024-02278-8

**Published:** 2025-01-24

**Authors:** Nisansala Chandimali, Seon Gyeong Bak, Eun Hyun Park, Hyung-Jin Lim, Yeong-Seon Won, Eun-Kyung Kim, Sang-Ik Park, Seung Jae Lee

**Affiliations:** 1 Functional Biomaterial Research Center, Korea Research Institute of Bioscience and Biotechnology (KRIBB), Jeongeup, 56212 Korea; 2 Applied Biological Engineering, KRIBB School of Biotechnology, University of Science and Technology, Daejeon, 34113 Korea; 3 Department of Veterinary Pathology, College of Veterinary Medicine and BK21 FOUR Program, Chonnam National University, Gwangju, 61186 Korea; 4 Scripps Korea Antibody Institute, Chuncheon, 24341 Korea; 5 Division of Research Management, Department of Bioresource Industrialization, Honam National Institute of Biological Resource, Mokpo, 58762 Korea; 6 Nutritional Education Major, Graduate School of Education, Dong-A University, Busan, 49315 Korea

**Keywords:** Diseases, Apoptosis

## Abstract

Free radicals, characterized by the presence of unpaired electrons, are highly reactive species that play a significant role in human health. These molecules can be generated through various endogenous processes, such as mitochondrial respiration and immune cell activation, as well as exogenous sources, including radiation, pollution, and smoking. While free radicals are essential for certain physiological processes, such as cell signaling and immune defense, their overproduction can disrupt the delicate balance between oxidants and antioxidants, leading to oxidative stress. Oxidative stress results in the damage of critical biomolecules like DNA, proteins, and lipids, contributing to the pathogenesis of various diseases. Chronic conditions such as cancer, cardiovascular diseases, neurodegenerative disorders, and inflammatory diseases have been strongly associated with the harmful effects of free radicals. This review provides a comprehensive overview of the characteristics and types of free radicals, their mechanisms of formation, and biological impacts. Additionally, we explore natural compounds and extracts studied for their antioxidant properties, offering potential therapeutic avenues for managing free radical-induced damage. Future research directions are also discussed to advance our understanding and treatment of free radical-associated diseases.

## Facts

- Free radicals cause oxidative stress, which contributes to the development of chronic diseases.
- Oxidative stress is strongly associated with cardiovascular diseases, neurodegenerative disorders, cancer, and diabetes.
- Antioxidants play a key role in neutralizing free radicals and mitigating oxidative stress.
- Unmanaged free radicals damage lipids, proteins, and DNA, leading to cellular dysfunction.

## Open questions

- What are the most effective natural compounds or extracts for targeting specific free radical-induced diseases?
- How can we optimize the bioavailability and stability of natural antioxidants to enhance their therapeutic efficacy in managing oxidative stress?
- What are the long-term effects and risks of antioxidant therapies?

## Introduction

Free radicals are atoms or molecules that possess one or more unpaired electrons, rendering them highly reactive and unstable. These reactive oxygen species (ROS) and reactive nitrogen species (RNS) are produced both endogenously and exogenously [1]. Endogenously, free radicals are generated as natural by-products of metabolic processes, such as mitochondrial electron transport, enzymatic reactions, and immune responses [2]. Exogenously, they arise from exposure to environmental factors like ultraviolet (UV) radiation, pollution, tobacco smoke, and industrial chemicals [3].

The dual nature of free radicals plays a crucial role in human physiology and pathology. On the one hand, they are vital for normal cellular functions. For instance, free radicals are involved in cell signaling pathways that regulate vascular tone, immune response, and apoptosis. Nitric oxide (NO), a reactive nitrogen species, acts as a signaling molecule that modulates vasodilation and neurotransmission [4, 5]. Similarly, the production of ROS by immune cells is essential for the destruction of invading pathogens [6].

However, an imbalance between free radical production and the body’s ability to detoxify them or repair the resulting damage leads to oxidative stress [7]. This imbalance can result from increased free radical production, decreased antioxidant defenses, or both [7]. Oxidative stress is detrimental as free radicals react with vital cellular components, including lipids, proteins, and DNA [8]. Lipid peroxidation disrupts cell membranes, protein oxidation leads to loss of function and structural integrity, and DNA damage can cause mutations and genomic instability [9].

The link between oxidative stress and disease pathogenesis is well-studied. In cardiovascular diseases, oxidative stress contributes to endothelial dysfunction, inflammation, and atherosclerosis [10]. In neurodegenerative diseases like Alzheimer’s and Parkinson’s, oxidative damage to neurons results in impaired function and cell death [11]. Cancer is associated with oxidative DNA damage that can initiate tumorigenesis [12]. Chronic inflammation, seen in conditions like rheumatoid arthritis and inflammatory bowel disease, is both a cause and consequence of sustained oxidative stress [13].

To counteract the harmful effects of free radicals, organisms have evolved complex antioxidant defense mechanisms [14]. These include enzymatic antioxidants such as superoxide dismutase (SOD), catalase, and glutathione peroxidase, which neutralize ROS, and non-enzymatic antioxidants such as vitamin C, vitamin E, and glutathione, which scavenge free radicals [15]. Additionally, dietary antioxidants derived from fruits, vegetables, and other plant sources play a significant role in maintaining the redox balance [16].

In recent years, interest in the therapeutic potential of natural compounds and plant extracts with antioxidant properties has grown [17]. Compounds such as phenolics, flavonoids, and carotenoids have been shown to mitigate oxidative damage and reduce the risk of chronic diseases. Research into these natural antioxidants offers promising avenues for preventive and therapeutic strategies against oxidative stress-related diseases [18]. However, high-dose antioxidant supplementation, even from natural sources, may pose risks. In some cases, it can exert pro-oxidant effects, potentially worsening oxidative stress. Excessive levels of vitamins C or E, for instance, may disrupt redox balance, impair normal cellular signaling, or even promote tumorigenesis [19]. Additionally, such doses might reduce the efficacy of chemotherapy or radiotherapy by counteracting the oxidative stress needed to target cancer cells [20]. Further research is essential to determine safe and effective dosing in clinical applications. Understanding the dual role of free radicals in health and disease is crucial for developing effective interventions. While antioxidant defenses of the body are robust, factors like lifestyle, diet, and environmental exposures significantly influence the balance between free radical production and antioxidant capacity. Advancements in this field could lead to novel therapies that harness natural antioxidants to combat oxidative stress and improve human health.

## Characteristics of free radicals

**Fig. 1:**
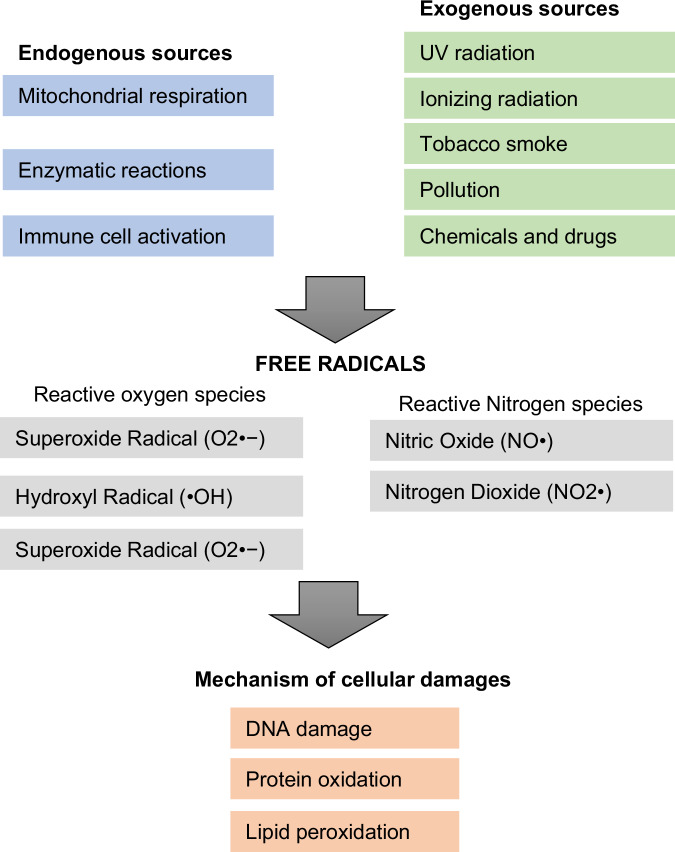
Overview of free radicals and their characteristics. Characteristics, sources, types, mechanisms of action, and antioxidant defenses related to free radicals. Free radicals, characterized by unpaired electrons, are highly reactive species generated endogenously through processes like mitochondrial respiration and enzymatic reactions, and exogenously from environmental factors such as UV radiation and pollution. The primary types include reactive oxygen species (ROS) and reactive nitrogen species (RNS). Free radicals induce cellular damage through lipid peroxidation, protein oxidation, and DNA damage, contributing to various diseases.

Free radicals are molecules or ions with unpaired electrons in their outermost electron shell, which makes them highly reactive and short-lived. The presence of these unpaired electrons drives free radicals to seek stability by pairing these electrons, often initiating chain reactions that can lead to significant cellular damage [3]. Free radicals can be generated through endogenous processes within the body or via exogenous sources. Endogenously, free radicals are by-products of normal cellular metabolism (Fig. 1) [3, 21]. For instance, during mitochondrial respiration, the electron transport chain occasionally leaks electrons that react with molecular oxygen to form superoxide radicals (O2•−) [22]. Enzymatic reactions, such as those involving cytochrome P450, xanthine oxidase, and nitric oxide synthase, can also produce free radicals [14]. Additionally, immune cells such as macrophages and neutrophils produce free radicals to combat pathogens in a process known as the respiratory burst [23]. Exogenous sources of free radicals include environmental factors such as ultraviolet (UV) radiation, ionizing radiation, tobacco smoke, pollution, and exposure to certain chemicals and drugs [24].

The primary types of free radicals include ROS and RNS [3]. ROS are oxygen-containing free radicals that play crucial roles in both physiological and pathological processes. Key examples of ROS include the superoxide radical (O2•−), hydroxyl radical (•OH), and peroxyl radical (RO2•) [25]. The superoxide radical is the precursor of most ROS and is produced primarily in the mitochondria. Although it is relatively weak in terms of reactivity, it can undergo dismutation, either spontaneously or catalyzed by the enzyme superoxide dismutase (SOD), to form hydrogen peroxide (H2O2) [2, 26]. H2O2, although not a radical, can be converted into the highly reactive hydroxyl radical through Fenton or Haber-Weiss reactions, particularly in the presence of transition metals such as iron or copper [27]. The hydroxyl radical (•OH) is the most reactive ROS and can indiscriminately react with and damage almost all types of biomolecules, including lipids, proteins, and nucleic acids. This extreme reactivity is due to its small size and lack of charge, allowing rapid diffusion across cellular structures. Unlike other ROS, it directly attacks biomolecules, causing extensive cellular damage through processes such as lipid peroxidation and DNA strand breaks [28, 29].

RNS, on the other hand, includes nitrogen-containing radicals such as nitric oxide (NO•) and nitrogen dioxide (NO2•). Nitric oxide is synthesized by nitric oxide synthases (NOS) and plays vital roles in vascular regulation, neurotransmission, and immune responses [5, 30]. Despite its beneficial roles, excessive production of NO• can lead to its reaction with superoxide to form peroxynitrite (ONOO − ), a potent oxidizing and nitrating agent [31]. ONOO− can cause nitration and oxidation of tyrosine residues in proteins, leading to altered protein function and signaling [32].

The mechanisms by which free radicals exert their effects involve several pathways. One of the primary mechanisms is lipid peroxidation, where free radicals attack polyunsaturated fatty acids in cell membranes [9]. This process begins with the abstraction of a hydrogen atom from the fatty acid, forming a lipid radical. The lipid radical reacts with molecular oxygen to form a lipid peroxyl radical, which can further propagate the chain reaction by abstracting hydrogen atoms from neighboring fatty acids, leading to a cascade of lipid peroxidation [9]. The end products of lipid peroxidation, such as malondialdehyde (MDA) and 4-hydroxynonenal (4-HNE), are highly reactive and can form adducts with proteins and DNA, impairing their function [33]. MDA, a stable end product of polyunsaturated fatty acid (PUFA) decomposition, is measured as an indicator of lipid peroxidation. Elevated levels of MDA have been linked to diseases like cardiovascular conditions, cancer, and neurodegenerative disorders [34]. Similarly, 4-HNE, a highly reactive aldehyde formed during lipid peroxidation, has been implicated in inflammation and aging. Both biomarkers are reliable markers for evaluating oxidative damage in cells, tissues, and biofluids, such as plasma and urine. These biomarkers are also used to track the efficacy of antioxidant therapies, with decreasing levels of MDA and 4-HNE indicating effective intervention [35].

Protein oxidation is another critical mechanism where free radicals modify amino acid side chains, form protein-protein cross-links, and fragment peptide chains. Such modifications can result in loss of enzymatic activity, altered cellular signaling, and degradation of structural proteins, contributing to cellular dysfunction and death [36, 37]. Specific amino acids, such as cysteine and methionine, are particularly susceptible to oxidation, which can disrupt the redox-sensitive signaling pathways and protein function [38].

DNA damage induced by free radicals includes base modifications, strand breaks, and cross-linking. The hydroxyl radical is especially notorious for causing DNA damage. It can abstract hydrogen atoms from the deoxyribose sugar backbone, leading to strand breaks, or add to the double bonds of DNA bases, resulting in base modifications such as 8-hydroxydeoxyguanosine (8-OHdG) [28]. These modifications can lead to mutations, genomic instability, and if not adequately repaired, contribute to carcinogenesis and other genetic disorders [39].

The body has evolved intricate antioxidant defense mechanisms to mitigate the damage caused by free radicals. Enzymatic antioxidants such as superoxide dismutase (SOD), catalase, and glutathione peroxidase play critical roles in neutralizing ROS [40]. SOD catalyzes the dismutation of superoxide into oxygen and hydrogen peroxide, which is then decomposed into water and oxygen by catalase and glutathione peroxidase [41]. Non-enzymatic antioxidants, including vitamins C and E, glutathione, and flavonoids, scavenge free radicals directly [15]. Vitamin E, a lipid-soluble antioxidant, protects cell membranes from lipid peroxidation [42], while vitamin C, a water-soluble antioxidant, can regenerate vitamin E from its oxidized form and scavenge various ROS [43].

## Mechanisms of free radical formation

Free radicals are formed through various mechanisms, both endogenous and exogenous, and understanding these processes is crucial for elucidating their role in health and disease (Table 1) [8]. These mechanisms involve a range of biochemical reactions and environmental interactions that generate ROS and RNS. One of the primary endogenous sources of free radicals is the mitochondrial electron transport chain (ETC) [3]. During cellular respiration, electrons are transferred through a series of complexes in the inner mitochondrial membrane to ultimately reduce oxygen to water. However, this process is not entirely efficient; a small percentage of electrons can prematurely leak and react with molecular oxygen, forming superoxide radicals (O2•−). This formation typically occurs at complexes I and III of the ETC [3]. The superoxide radical can be further converted to H2O2 by the enzyme SOD [44]. H2O2, although not a free radical itself, can generate highly reactive hydroxyl radicals (•OH) via Fenton reactions in the presence of transition metals like iron and copper [45]. This series of reactions highlights how normal cellular respiration inadvertently contributes to oxidative stress.

**Table 1:** Free radical formation mechanisms.

Mechanism	Source type	Description
Mitochondrial ETC	Endogenous	Leakage of electrons at complexes I and III forming superoxide radicals (O2•−).
NADPH Oxidase	Endogenous	Produces superoxide radicals in immune cells during respiratory burst.
Xanthine Oxidase	Endogenous	Generates superoxide and hydrogen peroxide during purine metabolism, especially under stress.
Nitric Oxide Synthase	Endogenous	Produces nitric oxide (NO•), which can form peroxynitrite (ONOO − ) with superoxide.
Lipid Peroxidation	Endogenous	Free radicals abstract hydrogen from polyunsaturated fatty acids, creating lipid radicals and peroxyl radicals.
Cytochrome P450	Endogenous	Leaks electrons to oxygen during metabolism, forming superoxide radicals.
Peroxisomes	Endogenous	Generate hydrogen peroxide through enzymatic actions such as acyl-CoA oxidase.
UV Radiation	Exogenous	Direct ionization causing the formation of ROS such as singlet oxygen and superoxide radicals.
Ionizing Radiation	Exogenous	High-energy particles ionize molecules, creating ROS and extensive cellular damage.
Environmental Pollutants	Exogenous	Cigarette smoke and heavy metals induce ROS through various mechanisms.
Drugs and Chemicals	Exogenous	Agents like doxorubicin and acetaminophen generate ROS as part of their action, causing oxidative stress.
Inflammatory Processes	Exogenous	Activated immune cells produce large amounts of ROS and RNS to combat pathogens.

Summary of endogenous and exogenous mechanisms of free radical formation and their effects on biomolecules. Endogenous sources include mitochondrial electron transport chain (ETC) leakage, enzymatic activities of NADPH oxidase, xanthine oxidase, nitric oxide synthase (NOS), cytochrome P450, and peroxisomes, along with lipid peroxidation and inflammation. Exogenous sources comprise ultraviolet (UV) radiation, ionizing radiation, environmental pollutants, and certain drugs and chemicals. The table outlines the specific free radicals produced by each mechanism and their consequent biological effects, such as DNA damage, lipid peroxidation, and protein oxidation, which contribute to oxidative stress and cellular damage.

Another significant endogenous mechanism is the activity of various oxidase enzymes. For example, NADPH oxidase, present in the membranes of phagocytic cells like neutrophils and macrophages, plays a critical role in the immune response [46]. Upon activation, NADPH oxidase transfers electrons from NADPH to oxygen, producing superoxide radicals [46]. This respiratory burst is essential for the destruction of pathogens [47]. Similarly, xanthine oxidase, an enzyme involved in purine metabolism, can generate superoxide and hydrogen peroxide as by-products, particularly under conditions of oxidative stress or tissue damage [48].

The enzyme nitric oxide synthase (NOS) is responsible for the production of nitric oxide (NO•), a reactive nitrogen species [49]. There are three isoforms of NOS: neuronal (nNOS), endothelial (eNOS), and inducible (iNOS) [50]. These enzymes catalyze the conversion of L-arginine to L-citrulline, releasing NO• in the process [49]. Nitric oxide serves various physiological functions, including vasodilation and neurotransmission [51]. However, in pathological conditions, excess NO• can react with superoxide to form ONOO − , a potent oxidant that can nitrate tyrosine residues in proteins and damage lipids and DNA [52].

Lipid peroxidation is another endogenous pathway that contributes to free radical formation. It begins with the abstraction of a hydrogen atom from a polyunsaturated fatty acid by a free radical, forming a lipid radical (L•) [9]. This lipid radical reacts with molecular oxygen to form a lipid peroxyl radical (LOO•), which can propagate the chain reaction by abstracting hydrogen atoms from neighboring lipids, leading to the generation of more lipid radicals and peroxyl radicals [9]. The end products of lipid peroxidation, such as malondialdehyde (MDA) and 4-hydroxynonenal (4-HNE), are themselves reactive and can further propagate oxidative damage [33].

Exogenous sources also contribute significantly to free radical formation [3]. Ultraviolet (UV) radiation from the sun can cause direct ionization of cellular molecules, leading to the formation of free radicals [53]. UV radiation can excite skin cells, resulting in the generation of ROS such as singlet oxygen (1O2) and superoxide radicals [53]. Ionizing radiation, such as X-rays and gamma rays, has enough energy to remove tightly bound electrons from atoms, creating ions and free radicals. These high-energy particles can directly break chemical bonds and form ROS, leading to extensive cellular damage [54].

Environmental pollutants and toxins are notable exogenous sources of free radicals [3]. For instance, cigarette smoke contains a complex mixture of free radicals and pro-oxidants. The tar phase of cigarette smoke contains long-lived radicals, while the gas phase contains short-lived radicals such as nitric oxide and superoxide. These radicals can diffuse into tissues and initiate chain reactions of lipid peroxidation and other oxidative processes [55]. Similarly, exposure to heavy metals such as lead, cadmium, and mercury can disrupt redox balance by catalyzing the production of ROS through Fenton-like reactions or by depleting cellular antioxidants [40].

Certain drugs and chemicals can also induce free radical formation [8]. For example, the chemotherapeutic agent doxorubicin generates ROS as part of its mechanism of action, contributing to its cytotoxic effects. However, this also leads to oxidative damage in non-cancerous tissues, causing side effects like cardiotoxicity [56]. Similarly, acetaminophen overdose leads to the formation of a toxic metabolite, N-acetyl-p-benzoquinone imine (NAPQI), which depletes glutathione and induces oxidative stress in the liver [57].

In addition to these sources, inflammatory processes in the body are significant contributors to free radical production [8]. During inflammation, activated immune cells such as neutrophils, macrophages, and eosinophils produce large amounts of ROS and RNS to combat pathogens [26]. This process involves enzymes like NADPH oxidase and myeloperoxidase, which produce superoxide and hypochlorous acid (HOCl), respectively. While these radicals are essential for pathogen clearance, excessive or chronic inflammation can lead to tissue damage due to the persistent high levels of free radicals [58].

Enzymatic activities also play a critical role in modulating the levels of free radicals in the body [59]. The enzyme cytochrome P450, involved in the metabolism of xenobiotics and endogenous compounds, can leak electrons to oxygen during its catalytic cycle, producing superoxide radicals [60]. Moreover, peroxisomes, organelles involved in lipid metabolism, contain enzymes such as acyl-CoA oxidase that generate hydrogen peroxide as a by-product [61].

## Diseases associated with free radicals

Free radicals, despite their essential roles in various physiological processes, can cause significant harm when their levels are not properly regulated [3]. An imbalance between free radical production and the body’s antioxidant defenses leads to oxidative stress, which is implicated in the pathogenesis of numerous diseases (Table 2) [8]. Excessive free radicals can damage cellular components such as lipids, proteins, and DNA, contributing to chronic conditions like cardiovascular diseases, neurodegenerative disorders, cancer, and inflammatory diseases [8, 21]. Conversely, insufficient free radical activity can impair the immune response and disrupt cellular signaling [14].

**Table 2:** Diseases associated with free radicals.

Disease / disease progression	Mechanism	Free radicals involved	Effects on biomolecules
**Cardiovascular diseases**			
Atherosclerosis	Endothelial dysfunction, oxidation of LDL, inflammation	Superoxide (O2• − ), NO•, ONOO−	Endothelial cell damage, formation of oxLDL, foam cell formation, plaque instability
Hypertension	Endothelial dysfunction, increased vascular resistance, altered renal function	Superoxide (O2• − ), ROS	Impaired vasodilation, damage to endothelial cells, sodium retention, increased blood volume
Heart Failure	Cardiomyocyte damage, mitochondrial dysfunction, fibrosis	ROS, superoxide (O2•−)	Cardiomyocyte apoptosis, mitochondrial damage, reduced ATP production, myocardial fibrosis
Myocardial Infarction	Ischemia-reperfusion injury	ROS, superoxide (O2•−)	Mitochondrial dysfunction, activation of inflammatory pathways, oxidative damage to cardiomyocytes damage to cardiomyocytes
**Cancer**			
Cancer Initiation and Progression	Direct DNA damage, activation of signaling pathways, tumor microenvironment	ROS, RNS	DNA mutations (e.g., 8-OHdG), activation of NF-κB and MAPKs, HIF-1 stabilization, genomic instability
Tumor Microenvironment	Hypoxia, inflammation, high metabolic activity	ROS	Promotion of angiogenesis, invasion, metastasis, immune evasion, enhanced VEGF and MMP expression
Metastasis	Epithelial-mesenchymal transition (EMT), invasion, survival in circulation	ROS	Loss of cell-cell adhesion, increased migration and invasion, enhanced expression of adhesion molecules
Immune Evasion	Suppression of anti-tumor immune response	ROS	Apoptosis of cytotoxic T cells and NK cells, recruitment of immunosuppressive cells (Tregs, MDSCs)
**Autoimmune diseases**			
Rheumatoid Arthritis (RA)	Oxidative modification of self-antigens, chronic inflammation	ROS, RNS	Formation of neo-antigens, activation of autoreactive T cells, joint inflammation and damage
Systemic Lupus Erythematosus (SLE)	Mitochondrial dysfunction, activation of type I IFN pathway	ROS, RNS	Generation of autoantibodies, chronic activation of type I IFN, immune dysregulation
Multiple Sclerosis (MS)	Demyelination, neuroinflammation, mitochondrial dysfunction	ROS	Myelin damage, neuronal damage, activation of microglia and astrocytes, promotion of neurodegeneration
Neurodegenerative Disorders			
Alzheimer’s Disease (AD)	ROS induced damage, Aβ aggregation, Impaired Aβ clearance mechanisms	ROS, RNS	Damage to lipids, proteins, and DNA, Aggregation of amyloid-beta (Aβ), Impaired autophagy and proteasomal degradation
Parkinson’s Disease (PD)	Dopamine metabolism, Mitochondrial dysfunction, Misfolding of alpha-synuclein, Neuroinflammation	hydroxyl radicals, Hydrogen Peroxide (H_2_O_2_)	Damage to lipids, proteins, and nucleic acids, Aggregation of alpha-synuclein, Impaired mitochondrial function, Neuroinflammation
Huntington’s Disease (HD)	Disruption of mitochondrial function, Impaired antioxidant defenses, Increased ROS production	Superoxide, RNS	Increased oxidative damage to motor neurons, Elevated oxidative damage markers, Impaired axonal transport
**Diabetes mellitus and its complications**			
Diabetes Mellitus	Hyperglycemia-induced ROS production, Formation of advanced glycation end products, Mitochondrial dysfunction	ROS, RNS	Cellular dysfunction and damage, Lipid peroxidation, Increased formation of AGEs, Mitochondrial damage, Impaired cellular energy production
Diabetic Neuropathy	Elevated ROS levels, Polyol pathway activity, Decreased NADPH and GSH, Osmotic damage	ROS, RNS	Lipid peroxidation, Damage to neuronal membranes, Impaired protein and DNA function, Osmotic damage to nerve cells
Diabetic Nephropathy	Increased ROS in renal cells, Activation of inflammatory pathways, Fibrosis and hypertrophy	Superoxide, H2O2	Lipid, protein, and DNA damage, Glomerular hypertrophy, Mesangial expansion, Tubulointerstitial fibrosis, Activation of NF-κB
Diabetic Retinopathy	Elevated ROS levels, Accumulation of AGEs, Activation of HIF-1α and VEGF	ROS, RNS	Damage to retinal endothelial cells, Increased vascular permeability, Formation of retinal neovascularization, Inflammatory responses triggered by RAGE
Cardiovascular Diseases	Oxidative damage to endothelial cells, Oxidation of LDL cholesterol, Impaired nitric oxide (NO) bioavailability	Superoxide, H_2_O_2_	Endothelial dysfunction, Formation of oxidized LDL, Reduced vasodilation, Increased vascular stiffness

Summary of the role of oxidative stress in various diseases. It outlines the primary mechanisms by which oxidative stress contributes to each condition, specifies the types of free radicals involved, and describes the effects on biomolecules such as lipids, proteins, and DNA.

## Cardiovascular diseases

Cardiovascular diseases (CVDs) are among the most prevalent and deadly health conditions worldwide, with oxidative stress playing a significant role in their pathogenesis [62]. Free radicals, particularly ROS and RNS, contribute to the development and progression of various cardiovascular conditions, including atherosclerosis, hypertension, heart failure, and myocardial infarction [3, 63].

## Atherosclerosis

Atherosclerosis is a chronic inflammatory disease characterized by the buildup of plaques within the arterial walls. This process begins with endothelial dysfunction, a critical event in the pathogenesis of atherosclerosis [64]. The endothelium, the inner lining of blood vessels, plays a vital role in maintaining vascular homeostasis by regulating vasodilation, blood flow, and platelet aggregation [65]. Endothelial cells produce nitric oxide (NO•), a free radical that acts as a vasodilator and inhibits platelet aggregation and adhesion [66].

However, under conditions of oxidative stress, superoxide radicals (O2•−) react with NO• to form ONOO − , reducing the bioavailability of NO• and leading to endothelial dysfunction [67]. This imbalance impairs vasodilation, promotes vasoconstriction, and increases vascular permeability, creating a conducive environment for the infiltration of lipoproteins and immune cells into the arterial wall. The oxidative modification of low-density lipoprotein (LDL) is another critical event in the development of atherosclerosis [68]. Free radicals can oxidize LDL particles, resulting in the formation of oxidized LDL (oxLDL) [69]. OxLDL is taken up by macrophages through scavenger receptors, leading to the formation of foam cells. These foam cells accumulate within the arterial intima, forming fatty streaks—the earliest visible lesions of atherosclerosis [70].

OxLDL also plays a pro-inflammatory role by stimulating endothelial cells to express adhesion molecules, such as vascular cell adhesion molecule-1 (VCAM-1) and intercellular adhesion molecule-1 (ICAM-1) [71]. These adhesion molecules facilitate the attachment and migration of monocytes into the arterial wall, where they differentiate into macrophages and contribute to the chronic inflammatory process [72]. Furthermore, oxLDL induces the release of pro-inflammatory cytokines, perpetuating inflammation and promoting the progression of atherosclerotic plaques [73].

As plaques grow, they can become unstable and prone to rupture. Plaque rupture exposes pro-thrombotic material to the bloodstream, triggering the formation of blood clots that can occlude arteries and lead to acute cardiovascular events such as myocardial infarction and stroke [74]. The oxidative stress-induced weakening of the fibrous cap covering the plaque, due to the degradation of extracellular matrix components by matrix metalloproteinases (MMPs), is a key factor in plaque instability [75].

## Hypertension

Hypertension, or high blood pressure, is another major cardiovascular condition linked to oxidative stress [76]. Free radicals contribute to hypertension through several mechanisms, including endothelial dysfunction, increased vascular resistance, and altered renal function [77]. As previously mentioned, the reaction of superoxide radicals with nitric oxide reduces NO• bioavailability, leading to impaired endothelium-dependent vasodilation. This results in increased vascular resistance and elevated blood pressure [67]. Additionally, free radicals can directly damage endothelial cells, further compromising their ability to regulate vascular tone [78].

Oxidative stress also affects the renin-angiotensin-aldosterone system (RAAS), a hormonal system that regulates blood pressure and fluid balance [79]. Angiotensin II (Ang II), a key component of RAAS, is known to stimulate the production of ROS, particularly in vascular smooth muscle cells and endothelial cells [80]. The increased ROS production induced by Ang II leads to vasoconstriction and promotes inflammation, contributing to the development and maintenance of hypertension [81]. Moreover, oxidative stress in the kidneys can impair their ability to regulate sodium excretion and blood volume. Free radicals can damage renal tubular cells and disrupt the function of sodium transporters, leading to sodium retention and increased blood volume, which further elevates blood pressure [82].

## Heart failure

Heart failure, a condition where the heart is unable to pump blood effectively, is also associated with oxidative stress [83]. Free radicals contribute to heart failure through direct damage to cardiomyocytes, mitochondrial dysfunction, and the promotion of fibrosis. Oxidative stress induces cardiomyocyte apoptosis (programmed cell death) and necrosis [84]. ROS can damage cellular components such as lipids, proteins, and DNA, leading to cell death and loss of cardiac contractile function. This damage is exacerbated by the fact that the heart has a high metabolic rate and oxygen demand, making it particularly vulnerable to oxidative injury [2].

Mitochondrial dysfunction is a critical factor in the progression of heart failure. Mitochondria are both a source and a target of ROS [85]. Under conditions of oxidative stress, the mitochondrial electron transport chain becomes less efficient, leading to increased production of superoxide radicals [86]. These radicals can further damage mitochondrial DNA, proteins, and lipids, impairing mitochondrial function and reducing ATP production, which is essential for cardiac muscle contraction [87].

Fibrosis, the excessive deposition of extracellular matrix proteins, is another hallmark of heart failure [88]. Free radicals stimulate the proliferation of cardiac fibroblasts and the production of collagen, contributing to myocardial fibrosis. This fibrosis stiffens the heart muscle, reducing its ability to contract and relax effectively, further impairing cardiac function [89].

## Myocardial infarction

Myocardial infarction (MI), commonly known as a heart attack, occurs when blood flow to a part of the heart muscle is blocked, leading to ischemia and tissue damage. Oxidative stress plays a crucial role in the pathogenesis of MI and the subsequent reperfusion injury that occurs when blood flow is restored [90].

During ischemia, the lack of oxygen and nutrients leads to a depletion of ATP and the accumulation of metabolic waste products, creating a highly stressful environment for cardiac cells. Upon reperfusion, the sudden influx of oxygen leads to a burst of ROS production, exacerbating cellular damage. This phenomenon, known as reperfusion injury, involves the oxidation of cellular components, mitochondrial dysfunction, and the activation of inflammatory pathways [91, 92]. Reperfusion injury is characterized by increased oxidative stress, which damages cardiomyocytes, endothelial cells, and the extracellular matrix [90]. The excessive ROS production during reperfusion can lead to the opening of the mitochondrial permeability transition pore (mPTP), causing mitochondrial swelling, loss of membrane potential, and cell death [2].

Inflammation also plays a significant role in reperfusion injury. ROS can activate pro-inflammatory signaling pathways, leading to the release of cytokines and the recruitment of immune cells to the site of injury [58]. The resulting inflammation exacerbates tissue damage and contributes to adverse remodeling of the heart, which can lead to heart failure if not properly managed [93].

## Cancer

Cancer is a complex and multifaceted disease characterized by uncontrolled cell growth, invasion into surrounding tissues, and often metastasis to distant organs [94]. The role of free radicals, particularly ROS and RNS, in the initiation, promotion, and progression of cancer is well-documented. These reactive species can damage DNA, proteins, and lipids, thereby contributing to genomic instability and tumor development [95].

While the body has evolved complex antioxidant systems to neutralize these reactive species, an imbalance between free radical production and antioxidant defenses can lead to oxidative stress, a state conducive to cancer development [8]. One of the primary mechanisms by which free radicals contribute to cancer is through direct DNA damage [96]. ROS and RNS can interact with DNA, causing base modifications, single- and double-strand breaks, and cross-linking. One common oxidative DNA lesion is 8-hydroxydeoxyguanosine (8-OHdG), which results from the interaction of ROS with guanine bases [97]. 8-OHdG can mispair with adenine during DNA replication, leading to G to T transversions. Such mutations, if they occur in critical genes like oncogenes or tumor suppressor genes, can drive the transformation of normal cells into malignant ones [98]. For example, mutations in the p53 tumor suppressor gene, often caused by oxidative stress, are frequently observed in various cancers [99].

Specially, in breast cancer, oxidative stress is linked to DNA damage and mutations in key oncogenes and tumor suppressor genes, such as BRCA1 and TP53. ROS can induce mutations in the BRCA1 gene, which is involved in DNA repair, leading to impaired DNA damage response and increased susceptibility to tumorigenesis [100]. In lung cancer, ROS generated by cigarette smoke cause DNA mutations and contribute to the activation of pro-inflammatory cytokines that promote tumorigenesis. Oxidative stress also induces the stabilization of HIF-1, enhancing angiogenesis and metastasis [101]. Furthermore, in colorectal cancer, ROS generated from inflammatory cells, such as neutrophils and macrophages, play a critical role in the progression of colorectal cancer by inducing DNA damage, promoting epithelial cell proliferation, and impairing apoptosis. Studies have shown that oxidative damage to the APC gene and activation of Wnt signaling, as a result of increased ROS levels, are key contributors to colorectal cancer pathogenesis [102].

In addition to direct DNA damage, free radicals also influence carcinogenesis through the modulation of cellular signaling pathways. ROS can act as secondary messengers in intracellular signaling cascades, affecting processes such as cell proliferation, apoptosis, and differentiation [103]. The activation of transcription factors like nuclear factor kappa B (NF-κB) and activator protein-1 (AP-1) by ROS is particularly significant in cancer. NF-κB, for instance, regulates the expression of genes involved in cell survival, inflammation, and immune responses [104]. Chronic activation of NF-κB by oxidative stress can promote tumor growth by enhancing cell proliferation and inhibiting apoptosis [105].

ROS-induced activation of mitogen-activated protein kinases (MAPKs) also plays a crucial role in cancer progression. MAPKs, including extracellular signal-regulated kinases (ERK), c-Jun N-terminal kinases (JNK), and p38 MAPKs, regulate various cellular activities such as growth, differentiation, and apoptosis. ROS can activate these kinases, leading to altered gene expression and promoting oncogenic transformation [106].

The tumor microenvironment, characterized by hypoxia, inflammation, and high metabolic activity, is another critical aspect of cancer influenced by oxidative stress [107]. Hypoxia, or low oxygen levels, is a common feature of solid tumors due to inadequate blood supply. Under hypoxic conditions, the stabilization of hypoxia-inducible factor-1 (HIF-1) occurs, a process heavily influenced by ROS. HIF-1 activation leads to the transcription of genes involved in angiogenesis, glycolysis, and cell survival, facilitating tumor growth and adaptation to hypoxic conditions [108]. ROS generated in the hypoxic tumor microenvironment further enhance the aggressive behavior of cancer cells by promoting angiogenesis, invasion, and metastasis [109].

Inflammation is closely linked to oxidative stress and cancer. Chronic inflammation, often driven by persistent infections, autoimmune diseases, or environmental factors, can create a pro-tumorigenic environment [110]. Inflammatory cells, such as macrophages and neutrophils, produce ROS and RNS as part of the immune response [58]. These reactive species can damage surrounding tissues and DNA, promoting mutations and genomic instability. Additionally, inflammatory cytokines and chemokines released by immune cells can activate signaling pathways that support tumor growth and survival [111]. For example, the cytokine tumor necrosis factor-alpha (TNF-α), produced during inflammation, can activate NF-κB and other pro-survival pathways in cancer cells [112].

Angiogenesis, the formation of new blood vessels, is essential for tumor growth and metastasis [113]. Free radicals play a pivotal role in the regulation of angiogenesis by modulating the expression of angiogenic factors like vascular endothelial growth factor (VEGF) [114]. ROS can induce the stabilization of HIF-1, which in turn upregulates VEGF expression, promoting the formation of new blood vessels to supply the growing tumor with nutrients and oxygen [115]. Furthermore, oxidative stress can enhance the expression of matrix metalloproteinases (MMPs), enzymes that degrade the extracellular matrix and facilitate tumor invasion and metastasis [116].

Metastasis, the spread of cancer cells from the primary tumor to distant sites, is a hallmark of cancer and a major cause of cancer-related mortality [117]. Oxidative stress contributes to metastasis by influencing multiple steps of the metastatic cascade, including epithelial-mesenchymal transition (EMT), invasion, intravasation, survival in the circulation, extravasation, and colonization of distant organs [118]. ROS can induce EMT, a process by which epithelial cells lose their cell-cell adhesion properties and gain migratory and invasive abilities. This transition is critical for the initial steps of metastasis [119]. Moreover, oxidative stress can enhance the expression of adhesion molecules and proteases, facilitating cancer cell detachment, invasion into surrounding tissues, and entry into the bloodstream [120].

The interaction between cancer cells and the immune system is another area where free radicals play a significant role. While the immune system can recognize and destroy cancer cells, tumors often develop mechanisms to evade immune surveillance [121]. Oxidative stress within the tumor microenvironment can suppress the anti-tumor immune response by inducing the apoptosis of cytotoxic T cells and natural killer (NK) cells, which are crucial for targeting and eliminating cancer cells [122]. Additionally, ROS can promote the recruitment and activation of immunosuppressive cells, such as regulatory T cells (Tregs) and myeloid-derived suppressor cells (MDSCs), which further inhibit the anti-tumor immune response and facilitate tumor progression [123].

Therapeutic strategies targeting oxidative stress in cancer are being explored, given the critical role of free radicals in tumor biology. Antioxidants, which neutralize ROS and RNS, have shown promise in preclinical studies for reducing oxidative damage and inhibiting cancer progression [122]. However, the use of antioxidants in cancer therapy is complex and context-dependent, as ROS also play essential roles in normal cellular signaling and immune responses. Furthermore, some cancer cells can develop resistance to oxidative stress, making antioxidant therapy less effective.

## Autoimmune diseases

Autoimmune diseases arise when the body’s immune system mistakenly attacks its own tissues, recognizing them as foreign. Free radicals, especially ROS and RNS, play a significant role in the pathogenesis of autoimmune diseases [124]. The generation of oxidative stress, characterized by an imbalance between the production of free radicals and the body’s antioxidant defenses, can lead to the activation and perpetuation of autoimmune responses [7]. Oxidative damage can initiate and propagate autoimmune reactions through several mechanisms.

One of the key mechanisms by which free radicals contribute to autoimmune diseases is through the modification of self-antigens [125]. Oxidative stress can induce structural changes in proteins and other molecules, creating neo-antigens that are recognized as foreign by the immune system [126]. For instance, ROS can induce the formation of carbonyl groups on proteins, altering their structure and antigenicity. These modified proteins can be taken up by antigen-presenting cells (APCs), processed, and presented to T cells, leading to the activation of autoreactive T cells and the production of autoantibodies. This mechanism is implicated in diseases such as rheumatoid arthritis (RA), where oxidative modifications of proteins in the synovial fluid contribute to the autoimmune response [127, 128].

In addition to modifying self-antigens, free radicals can directly damage tissues, triggering inflammation and immune activation [58]. Oxidative stress can lead to lipid peroxidation, a process in which ROS react with lipids in cell membranes, resulting in the formation of lipid peroxides and aldehydes such as malondialdehyde (MDA) and 4-hydroxynonenal (4-HNE) [9]. These reactive aldehydes can form adducts with proteins, further contributing to neo-antigen formation and immune activation. The resulting inflammation and tissue damage can perpetuate the autoimmune response, creating a vicious cycle of oxidative stress and immune-mediated tissue injury [129].

Mitochondrial dysfunction is another crucial aspect of the relationship between free radicals and autoimmune diseases. In autoimmune diseases such as systemic lupus erythematosus (SLE), mitochondrial damage and the release of mitochondrial DNA (mtDNA) into the cytoplasm and extracellular space have been observed [130]. Extracellular mtDNA can act as a damage-associated molecular pattern (DAMP), triggering the activation of innate immune receptors such as toll-like receptors (TLRs) and inducing the production of type I interferons (IFNs) [131]. The chronic activation of the type I IFN pathway is a hallmark of SLE and contributes to the dysregulation of immune responses in this disease [132].

The role of oxidative stress in the activation and function of immune cells is also critical in autoimmune diseases [133]. ROS can modulate the activity of various transcription factors and signaling pathways involved in immune cell activation, differentiation, and function. For example, ROS can activate nuclear factor kappa B (NF-κB), a key transcription factor that regulates the expression of pro-inflammatory cytokines, chemokines, and adhesion molecules [134]. Chronic activation of NF-κB by oxidative stress can lead to sustained inflammation and the perpetuation of autoimmune responses [135]. Additionally, ROS can influence the differentiation and function of T cells, promoting the polarization of T helper 17 (Th17) cells, which are known to play a pathogenic role in autoimmune diseases such as multiple sclerosis (MS) and psoriasis [136]. Oxidative stress is integral in driving Th17 cell differentiation by modulating key transcription factors, such as RORγt, and cytokines like IL-6, IL-23, and TGF-β [137]. In conditions such as MS, Th17-driven inflammation contributes to neurodegeneration and tissue damage [138]. The involvement of oxidative stress in the breakdown of immune tolerance is another crucial mechanism in the pathogenesis of autoimmune diseases. Immune tolerance mechanisms, including central tolerance in the thymus and peripheral tolerance in secondary lymphoid organs, are essential for preventing autoimmunity [139, 140]. Oxidative stress can disrupt these tolerance mechanisms by inducing apoptosis of regulatory T cells (Tregs), which are critical for maintaining immune homeostasis and preventing autoimmunity [141]. Additionally, ROS can impair the function of Tregs, reducing their ability to suppress autoreactive T cells and control immune responses. This disruption of immune tolerance contributes to the development and progression of autoimmune diseases [142].

Chronic inflammation, often driven by oxidative stress, is a common feature of autoimmune diseases. Inflammatory cytokines such as tumor necrosis factor-alpha (TNF-α), interleukin-1 beta (IL-1β), and interleukin-6 (IL-6) are elevated in many autoimmune diseases and contribute to the inflammatory milieu [143]. ROS can enhance the production of these cytokines by activating transcription factors like NF-κB and AP-1, creating a feed-forward loop that amplifies inflammation and tissue damage. The persistent inflammatory environment not only exacerbates oxidative stress but also promotes the recruitment and activation of additional immune cells, further perpetuating the autoimmune response [58].

One illustrative example of the role of free radicals in autoimmune disease is rheumatoid arthritis (RA). RA is characterized by chronic inflammation of the synovial joints, leading to joint damage and deformity. Oxidative stress plays a significant role in the pathogenesis of RA [144]. Elevated levels of ROS and oxidative damage markers, such as 8-OHdG and MDA, have been detected in the synovial fluid and tissues of RA patients. Oxidative modifications of synovial proteins contribute to the formation of neo-antigens, triggering autoimmune responses [145].

Another example is systemic lupus erythematosus (SLE), a systemic autoimmune disease characterized by the production of autoantibodies against nuclear antigens. Oxidative stress is implicated in the pathogenesis of SLE through multiple mechanisms, including mitochondrial dysfunction, activation of the type I IFN pathway, and the generation of oxidative DNA damage [130]. The chronic activation of the type I IFN pathway by oxidative stress contributes to the dysregulation of immune responses and the production of autoantibodies in SLE [146].

Multiple sclerosis (MS) is a chronic autoimmune disease affecting the central nervous system, characterized by demyelination and neurodegeneration. Oxidative stress plays a critical role in MS by promoting inflammation, mitochondrial dysfunction, and neurodegeneration [147]. Elevated levels of ROS and oxidative damage markers have been detected in the brains and cerebrospinal fluid of MS patients [148]. ROS can damage myelin and oligodendrocytes, the cells responsible for myelin production, leading to demyelination. Additionally, oxidative stress can activate microglia and astrocytes, contributing to neuroinflammation and further neuronal damage [136, 149].

## Neurodegenerative disorders

Neurodegenerative diseases, including Alzheimer’s disease (AD), Parkinson’s disease (PD), Huntington’s disease (HD), and amyotrophic lateral sclerosis (ALS), are characterized by the progressive loss of structure and function of neurons, leading to cognitive and motor impairments [150]. Increasing evidence points to oxidative stress, mediated by free radicals such as ROS and RNS, as a critical factor in the pathogenesis of these diseases [40]. The brain is particularly vulnerable to oxidative damage due to its high oxygen consumption, abundant lipid content, and relatively low antioxidant capacity [151].

In Alzheimer’s disease (AD), oxidative stress is a hallmark feature and plays a pivotal role in the disease’s pathogenesis. AD is characterized by the accumulation of amyloid-beta (Aβ) plaques and neurofibrillary tangles composed of hyperphosphorylated tau protein [152]. Oxidative stress can exacerbate these pathological features through several mechanisms. ROS can directly damage cellular macromolecules, including lipids, proteins, and DNA, leading to neuronal dysfunction and death [87]. Furthermore, oxidative stress can promote the aggregation of Aβ peptides. Aβ itself can generate ROS, creating a vicious cycle of oxidative stress and Aβ accumulation. The interaction between Aβ and metals like iron and copper can catalyze the production of ROS via Fenton reactions [153]. Additionally, oxidative stress can impair the clearance mechanisms of Aβ, including autophagy and proteasomal degradation, contributing to its accumulation [154].

Mitochondrial dysfunction is another critical aspect of AD pathogenesis linked to oxidative stress. Mitochondria are both a major source and target of ROS. In AD, mitochondrial abnormalities, including impaired electron transport chain function and reduced mitochondrial biogenesis, lead to increased ROS production and diminished ATP generation. The resulting energy deficit and oxidative damage to mitochondrial DNA (mtDNA) and proteins further compromise neuronal function and survival [155]. While oxidative stress is a major factor in mitochondrial dysfunction in AD, other contributing mechanisms also play a significant role. The accumulation of amyloid-beta (Aβ) plaques and the hyperphosphorylation of tau proteins contribute to mitochondrial abnormalities and exacerbate oxidative damage. Aβ peptides, in particular, have been shown to disrupt mitochondrial function by interacting with mitochondrial membranes and promoting the production of ROS [156]. Furthermore, tau aggregation can impair mitochondrial dynamics, leading to decreased mitochondrial motility and compromised mitochondrial function. This cascade of mitochondrial damage, along with the impaired ability of mitochondria to regulate calcium homeostasis and bioenergetics, leads to further neuronal injury and death [157]. Additionally, mitochondrial DNA mutations and reduced mitophagy exacerbate mitochondrial dysfunction and contribute to the progression of AD [158].

In Parkinson’s disease (PD), oxidative stress is closely associated with the degeneration of dopaminergic neurons in the substantia nigra. The primary pathological hallmark of PD is the presence of Lewy bodies, which are intracellular aggregates of the protein alpha-synuclein. Oxidative stress contributes to PD pathogenesis through multiple mechanisms, including mitochondrial dysfunction, protein misfolding, and neuroinflammation [159]. Dopaminergic neurons are particularly susceptible to oxidative stress due to the metabolism of dopamine. The enzymatic oxidation of dopamine generates ROS and dopamine quinones, which can react with cellular proteins, lipids, and nucleic acids, causing oxidative damage. Additionally, the metabolism of dopamine by monoamine oxidase (MAO) produces hydrogen peroxide (H_2_O_2_), which can be converted to highly reactive hydroxyl radicals via Fenton reactions in the presence of transition metals [160].

Mitochondrial dysfunction is a central feature of PD, with complex I of the electron transport chain being particularly affected. Impairment of complex I leads to increased ROS production and decreased ATP synthesis, contributing to neuronal energy deficits and cell death [161]. Alpha-synuclein, the primary component of Lewy bodies, is prone to oxidative modifications, which can promote its aggregation and toxicity. Oxidative stress can induce the misfolding and oligomerization of alpha-synuclein, contributing to its accumulation and the formation of toxic aggregates [162]. Additionally, the interaction between alpha-synuclein and phospholipids in cell membranes can generate ROS, further exacerbating oxidative damage and neuronal dysfunction [163]. Neuroinflammation, driven by the activation of microglia, is another critical factor in PD pathogenesis. Activated microglia produce ROS and pro-inflammatory cytokines, contributing to the oxidative and inflammatory environment in the PD brain. Chronic neuroinflammation can sustain oxidative stress and neuronal damage, creating a feedback loop that exacerbates disease progression [164].

In Huntington’s disease (HD), oxidative stress plays a significant role in the neurodegenerative process. HD is caused by an expanded CAG repeat in the huntingtin (HTT) gene, leading to the production of a mutant huntingtin protein with toxic gain-of-function properties. The mutant huntingtin protein can disrupt mitochondrial function and increase ROS production. Mitochondrial dysfunction in HD includes impaired electron transport chain activity, reduced mitochondrial biogenesis, and defective mitophagy, all of which contribute to increased oxidative stress and neuronal damage [165]. Oxidative damage to proteins, lipids, and DNA is evident in HD brain tissues, indicating the pervasive impact of oxidative stress. Additionally, the mutant huntingtin protein can impair the function of the transcription factor PGC-1α, which is crucial for the regulation of antioxidant defenses and mitochondrial biogenesis. The resulting decrease in antioxidant capacity further exacerbates oxidative stress and neuronal vulnerability in HD [166].

Amyotrophic lateral sclerosis (ALS) is another neurodegenerative disease where oxidative stress plays a central role. ALS is characterized by the progressive loss of motor neurons, leading to muscle weakness and paralysis. Mutations in the superoxide dismutase 1 (SOD1) gene are associated with familial forms of ALS [167]. SOD1 is an enzyme that catalyzes the dismutation of superoxide radicals into hydrogen peroxide and oxygen, providing a crucial antioxidant defense. Mutant SOD1 proteins can form toxic aggregates and lose their enzymatic activity, leading to increased oxidative stress [168]. In ALS, oxidative damage to motor neurons is a significant pathological feature. Elevated levels of oxidative damage markers, such as 8-OHdG and protein carbonyls, have been detected in ALS patients. Mitochondrial dysfunction, impaired axonal transport, and neuroinflammation are key contributors to oxidative stress in ALS [169]. Additionally, the activation of microglia and astrocytes in ALS leads to the production of ROS and pro-inflammatory cytokines, further exacerbating oxidative damage and motor neuron degeneration [170].

## Diabetes mellitus and its complications

Diabetes mellitus is a complex metabolic disorder characterized by chronic hyperglycemia resulting from defects in insulin production, insulin action, or both. This condition leads to a wide range of complications that significantly affect various organs and systems [171]. One of the critical factors underlying these complications is oxidative stress, which is primarily driven by free radicals such as ROS and RNS. The interplay between hyperglycemia and oxidative stress contributes to the pathogenesis of diabetic complications, including neuropathy, nephropathy, retinopathy, and cardiovascular diseases [172].

Hyperglycemia-induced oxidative stress begins with the excessive production of ROS due to elevated glucose levels. Glucose undergoes autoxidation, producing ROS as by-products [173]. Furthermore, high glucose levels increase the formation of advanced glycation end products (AGEs) through the non-enzymatic reaction between reducing sugars and cellular proteins, lipids, or nucleic acids. AGEs, in turn, generate ROS through various mechanisms, including metal-catalyzed oxidation reactions. This cascade of oxidative stress can lead to cellular dysfunction and damage, significantly impacting various tissues and organs [174, 175].

Mitochondrial dysfunction is another critical aspect of oxidative stress in diabetes. Mitochondria are central to cellular energy production, and their function is intimately linked with ROS generation. In the context of diabetes, chronic hyperglycemia overwhelms the mitochondrial electron transport chain, leading to increased ROS production [176]. This oxidative stress damages mitochondrial components, including lipids, proteins, and mitochondrial DNA (mtDNA) [177]. The resulting mitochondrial dysfunction further exacerbates oxidative stress and impairs cellular energy production, creating a vicious cycle of damage and dysfunction [178].

The impact of oxidative stress in diabetes is particularly evident in its complications. Diabetic neuropathy, a common complication, is characterized by nerve damage, particularly in the peripheral nervous system. Elevated ROS levels lead to lipid peroxidation, resulting in the formation of toxic aldehydes that damage neuronal membranes [179]. Additionally, oxidative stress impairs protein and DNA function, contributing to neuronal dysfunction and death. The polyol pathway, where excess glucose is converted to sorbitol and fructose by the enzyme aldose reductase, further exacerbates oxidative stress by depleting NADPH, a crucial cofactor for the regeneration of reduced glutathione (GSH) [180]. The resulting decrease in antioxidant capacity and increased sorbitol accumulation cause osmotic damage to nerve cells, worsening neuropathy [181].

Diabetic nephropathy, another major complication, involves progressive kidney damage that can lead to proteinuria, decreased glomerular filtration rate (GFR), and eventually end-stage renal disease [182]. In this context, oxidative stress plays a significant role by increasing ROS levels in renal cells, which damages lipids, proteins, and DNA. This oxidative damage activates signaling pathways that promote inflammation and fibrosis, leading to glomerular hypertrophy, mesangial expansion, and tubulointerstitial fibrosis [183]. Increased ROS also activate nuclear factor kappa B (NF-κB), which drives the expression of pro-inflammatory cytokines and adhesion molecules, contributing to renal dysfunction [134].

In diabetic retinopathy, a major microvascular complication affecting the retina, oxidative stress leads to damage of retinal endothelial cells. Elevated ROS levels increase vascular permeability and leakage of plasma components, contributing to the development of retinopathy [184]. AGEs accumulate in the retina and interact with their receptor, RAGE, triggering inflammatory responses and exacerbating oxidative damage [185]. Additionally, ROS activate the hypoxia-inducible factor-1 alpha (HIF-1α) pathway, leading to increased expression of vascular endothelial growth factor (VEGF) and the formation of retinal neovascularization [186].

Cardiovascular diseases, including coronary artery disease (CAD), stroke, and peripheral artery disease (PAD), are prevalent among individuals with diabetes. Oxidative stress contributes to these conditions by damaging endothelial cells and impairing their function [187]. Elevated ROS levels lead to endothelial dysfunction, which is a precursor to atherosclerosis. Oxidative modification of low-density lipoprotein (LDL) cholesterol forms oxidized LDL (oxLDL), which is highly atherogenic [68]. OxLDL promotes macrophage recruitment and activation in the arterial wall, contributing to plaque formation. Furthermore, oxidative stress impairs nitric oxide (NO) bioavailability, reducing vasodilation and increasing vascular stiffness [188].

## Role of antioxidants

Antioxidants play a crucial role in managing free radicals and mitigating oxidative stress, which is associated with a wide range of diseases. By neutralizing free radicals and reducing oxidative damage, antioxidants help maintain cellular integrity and function, ultimately contributing to overall health and disease prevention [14]. The body employs a multifaceted defense system to counteract oxidative stress, involving both endogenous and exogenous antioxidants [189]. Endogenous antioxidants are produced within the body and include enzymatic and non-enzymatic components. Key enzymatic antioxidants include SOD, catalase, and glutathione peroxidase [189]. SOD catalyzes the conversion of superoxide radicals into hydrogen peroxide, which is then further detoxified by catalase and glutathione peroxidase. These enzymes work in tandem to reduce the levels of harmful ROS and prevent oxidative damage [41]. Non-enzymatic antioxidants, such as glutathione (GSH), play a critical role in detoxifying ROS. GSH is a tripeptide that can directly neutralize free radicals and regenerate other antioxidants, maintaining the balance of oxidative stress within cells [15].

Despite the body’s robust antioxidant defenses, the increasing burden of oxidative stress due to factors like environmental pollutants, unhealthy diets, and chronic diseases often overwhelms these defenses. This is where exogenous antioxidants—obtained from dietary sources or supplements—become vital [24]. Exogenous antioxidants include vitamins, minerals, and phytochemicals that can directly scavenge free radicals or enhance the body’s own antioxidant defenses [190]. Among vitamins, vitamin C and vitamin E are prominent. Vitamin C, also known as ascorbic acid, is a water-soluble antioxidant that can neutralize ROS in the aqueous environments of the body, such as the cytoplasm and extracellular fluids. It also regenerates vitamin E, a fat-soluble antioxidant, enhancing its efficacy [191]. Vitamin E, primarily in the form of alpha-tocopherol, protects cellular membranes from oxidative damage by neutralizing lipid peroxyl radicals. Together, these vitamins work synergistically to protect against oxidative damage and support cellular health [192]. Moreover, Vitamins C and E enhance each other’s therapeutic potential through their synergistic effects. Vitamin C, a water-soluble antioxidant, helps regenerate oxidized vitamin E, allowing it to continue neutralizing lipid peroxyl radicals in cellular membranes. Meanwhile, vitamin C itself scavenges ROS in aqueous environments, particularly in the cytoplasm and extracellular fluids. This collaboration ensures a more comprehensive antioxidant defense, protecting various cellular structures from oxidative damage [193].

Phytochemicals, which are bioactive compounds found in plants, also contribute to antioxidant defenses. Flavonoids, carotenoids, and polyphenols are examples of phytochemicals with significant antioxidant properties [194]. Flavonoids, such as quercetin and catechins, are known for their ability to scavenge free radicals and modulate oxidative stress pathways [195]. Carotenoids, including beta-carotene, lutein, and zeaxanthin, provide protection against oxidative damage, particularly in eye health [196]. Polyphenols, found in foods like green tea and red wine, possess potent antioxidant activity and have been linked to reduced risks of chronic diseases [197].

The therapeutic potential of antioxidants extends to various health conditions. In cardiovascular diseases, antioxidants can help reduce oxidative stress and improve endothelial function, thus potentially preventing atherosclerosis and other heart-related issues [198]. In neurodegenerative diseases like Alzheimer’s and Parkinson’s, antioxidants may help protect neurons from oxidative damage and slow disease progression [199]. Furthermore, antioxidants have shown promise in managing diabetes-related complications by reducing oxidative stress and improving glycemic control [200].

## Natural extracts and compounds in managing free radicals

Natural extracts and compounds refer to substances derived from plants, fruits, vegetables, herbs, and other natural sources that possess bioactive properties beneficial for health. These natural compounds are often rich in antioxidants, which are capable of neutralizing free radicals and reducing oxidative stress [201]. They include a diverse range of phytochemicals that have been extensively studied for their potential therapeutic effects against oxidative damage and associated diseases (Table 3) [201]. One prominent category of natural extracts includes flavonoids, a diverse group of polyphenolic compounds found in fruits, vegetables, and beverages like tea. For example, quercetin, found in onions and apples, and catechins, present in green tea, are well-documented for their potent antioxidant activities [195]. Similarly, curcumin, derived from turmeric, is noted for its ability to scavenge free radicals and modulate inflammatory pathways [202]. Another significant group includes carotenoids such as beta-carotene, lutein, and zeaxanthin, which are found in colorful fruits and vegetables like carrots and spinach [203]. These compounds have been shown to protect against oxidative damage and support eye health.

**Table 3:** Natural antioxidant extracts and phenolic compounds.

Category	Compound	Source	Mechanism of action	Health benefits
Flavonoids	Quercetin	Onions, Apples, Berries	Scavenges free radicals, inhibits NF-κB	Reduces inflammation, protects against oxidative stress
	Catechins	Green Tea	Chelates metal ions, enhances SOD and catalase activity	Reduces oxidative stress, supports heart health
	Anthocyanins	Berries, Red Cabbage	Scavenges free radicals, modulates Nrf2 pathway	Protects against oxidative damage, supports eye health
Phenolic Acids	Caffeic Acid	Coffee, Certain Fruits	Scavenges free radicals, inhibits ROS production	Reduces inflammation, supports overall health
	Ferulic Acid	Grains, Vegetables	Scavenges free radicals, enhances glutathione peroxidase	Boosts internal antioxidant defenses
Tannins	General Tannins	Tea, Wine, Various Fruits	Scavenges free radicals, complexes with metal ions	Protects against oxidative damage, supports overall health
Other Polyphenols	Resveratrol	Grapes, Red Wine	Neutralizes free radicals, chelates metal ions, activates Nrf2	Cardiovascular benefits, neuroprotection
	Curcumin	Turmeric	Scavenges free radicals, inhibits ROS production, modulates NF-κB and Nrf2	Anti-inflammatory, supports joint and digestive health

Overview of various natural extracts and phenolic compounds, categorized into flavonoids, carotenoids, phenolic acids, tannins, and other polyphenols. It details specific compounds within each category, their natural sources (such as fruits, vegetables, herbs, and beverages), their mechanisms of action (including scavenging free radicals, chelating metal ions, enhancing endogenous antioxidants, and modulating inflammatory pathways), and their associated health benefits (such as cardiovascular health, neuroprotection, eye health, liver health, and general well-being).

In addition to flavonoids and carotenoids, other natural extracts with notable antioxidant properties include resveratrol, found in grapes and red wine, and silymarin, derived from milk thistle. Resveratrol has been associated with cardiovascular benefits and neuroprotection, while silymarin is known for its hepatoprotective effects [204]. Additionally, natural extracts from herbs such as ginseng and rosemary contain bioactive compounds with demonstrated antioxidant capabilities [205]. These extracts have been studied for their potential to mitigate oxidative stress and improve health outcomes in various conditions.

The growing interest in these natural extracts and compounds is driven by their potential as complementary or alternative therapies in managing diseases linked to oxidative stress. Research into these natural sources continues to reveal their diverse mechanisms of action, making them valuable components in the quest for effective strategies to combat oxidative damage and promote overall health.

## Phenolic compounds

Phenolic compounds, a diverse group of phytochemicals characterized by the presence of one or more hydroxyl groups attached to an aromatic ring, are renowned for their potent antioxidant properties. These compounds are widely distributed in the plant kingdom and have been extensively studied for their ability to manage free radicals and oxidative stress [206]. Phenolic compounds can be broadly categorized into flavonoids, phenolic acids, tannins, and other polyphenols, each with unique mechanisms of action in neutralizing free radicals [207].

Flavonoids, such as quercetin, catechins, and anthocyanins, are among the most studied phenolic compounds [195]. Quercetin, found in onions, apples, and berries, exerts its antioxidant effects primarily through its ability to scavenge free radicals directly [208]. The hydroxyl groups in quercetin’s structure can donate electrons to neutralize ROS like superoxide radicals and hydroxyl radicals. Additionally, quercetin modulates cellular signaling pathways involved in oxidative stress. It inhibits the activation of nuclear factor kappa B (NF-κB), a transcription factor that drives the expression of pro-inflammatory cytokines. By reducing NF-κB activation, quercetin decreases the production of inflammatory mediators, thereby mitigating oxidative damage [209].

Catechins, found predominantly in green tea, also display significant antioxidant activity. These compounds can chelate metal ions, such as iron and copper, which are involved in generating ROS through Fenton reactions. By sequestering these metal ions, catechins prevent the formation of hydroxyl radicals, thereby reducing oxidative stress. Catechins also enhance the activity of endogenous antioxidant enzymes like superoxide dismutase (SOD) and catalase, bolstering the body’s ability to neutralize ROS [210, 211].

Anthocyanins, responsible for the red, blue, and purple colors of many fruits and vegetables, possess potent antioxidant properties due to their ability to donate electrons and stabilize free radicals. These compounds also exhibit metal-chelating properties, similar to catechins, which further aids in reducing oxidative stress [212]. Studies have shown that anthocyanins can modulate signaling pathways involved in oxidative stress, including the Nrf2 pathway. Activation of Nrf2 leads to the upregulation of various antioxidant and detoxifying enzymes, enhancing the cellular defense against oxidative damage [213].

Phenolic acids, such as caffeic acid and ferulic acid, are another important class of phenolic compounds. Caffeic acid, found in coffee and certain fruits, is known for its ability to scavenge a range of free radicals, including superoxide and hydroxyl radicals [214]. Caffeic acid also exerts its antioxidant effects by inhibiting the production of ROS through the suppression of inflammatory pathways. It can modulate the activity of enzymes involved in the production of inflammatory mediators, such as lipoxygenase and cyclooxygenase, thus reducing oxidative stress and inflammation [215].

Ferulic acid, present in grains and vegetables, similarly scavenges free radicals and protects cellular components from oxidative damage. It has been shown to enhance the activity of endogenous antioxidants, including glutathione peroxidase and SOD. By boosting the body’s internal antioxidant defenses, ferulic acid contributes to a more effective response against oxidative stress [216]. Tannins, another class of phenolic compounds, are known for their strong antioxidant properties. Tannins can form complexes with metal ions, thereby preventing the Fenton reaction and reducing ROS generation [217]. They also possess the ability to scavenge free radicals directly. Tannins have been shown to reduce oxidative damage to lipids, proteins, and DNA, contributing to their protective effects against various diseases [217].

Other polyphenols, such as resveratrol and curcumin, also have significant antioxidant activity [218]. Resveratrol, found in grapes and red wine, is known for its ability to neutralize free radicals and chelate metal ions. Resveratrol activates the Nrf2 pathway, leading to increased expression of antioxidant enzymes and enhanced cellular protection against oxidative damage [219]. Curcumin, derived from turmeric, has been extensively studied for its antioxidant properties. It can scavenge free radicals, inhibit ROS production, and modulate various signaling pathways involved in oxidative stress. Curcumin’s ability to influence multiple cellular pathways, including NF-κB and Nrf2, underscores its potential as a therapeutic agent in managing oxidative stress and inflammation [220, 221].

## Future directions in research on free radicals and antioxidants

**Fig. 2:**
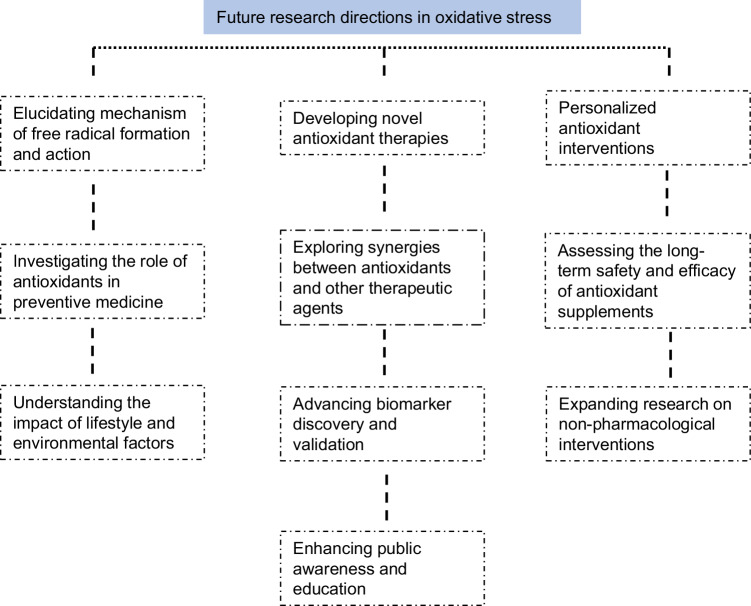
Future research directions in oxidative stress management. key research areas for advancing the management of oxidative stress. The central focus is on understanding and improving oxidative stress management through ten key avenues: elucidating mechanisms of free radical action, developing novel antioxidant therapies, personalizing antioxidant interventions, investigating antioxidants in preventive medicine, exploring synergies with other therapies, assessing long-term safety of supplements, understanding lifestyle and environmental impacts, advancing biomarker discovery, expanding non-pharmacological interventions, and enhancing public awareness and education. These areas collectively aim to deepen insights into oxidative stress and refine therapeutic strategies for better health outcomes.

As our understanding of free radicals and their role in disease pathology deepens, several promising avenues for future research emerge (Fig. 2). Addressing these research gaps and exploring new methodologies could enhance our ability to manage oxidative stress and develop more effective therapeutic strategies. While much is known about free radicals and their general effects, detailed mechanistic studies are needed to understand how specific radicals contribute to various diseases at the molecular level. Investigating the precise pathways through which free radicals induce cellular damage and interact with biological macromolecules will provide deeper insights into their roles in disease processes.

Research into new antioxidant compounds and natural extracts is crucial for discovering more effective therapeutic agents. This includes exploring lesser-known antioxidants, optimizing the delivery and bioavailability of existing antioxidants, and developing synthetic analogs with enhanced efficacy. The goal is to create targeted therapies that can specifically neutralize harmful radicals without interfering with essential physiological processes. Given the variability in individual responses to antioxidants, future research should focus on personalized approaches to antioxidant therapy. This involves understanding genetic, environmental, and lifestyle factors that influence individual oxidative stress levels and responses to antioxidants. Developing personalized nutritional and pharmacological interventions based on these factors could enhance therapeutic outcomes and reduce side effects.

In developing personalized antioxidant therapies, several challenges must be considered. Genetic variability plays a significant role, as differences in genes associated with oxidative stress response can impact how individuals metabolize antioxidants. Moreover, the bioavailability and absorption of antioxidants vary between individuals, which complicates dosage recommendations. Environmental and lifestyle factors, including diet, exercise, and exposure to pollutants, also influence oxidative stress levels, making it difficult to develop standardized treatments. Additionally, the safety and potential interactions of high-dose antioxidants with other medications pose challenges in tailoring antioxidant therapy for specific individuals.

Expanding research on the preventive effects of antioxidants in populations at risk for chronic diseases can provide valuable insights into their potential as preventive agents. Clinical trials evaluating the impact of antioxidant-rich diets or supplements on disease incidence, progression, and overall health outcomes will be essential in establishing their efficacy and safety. Combining antioxidants with other therapeutic agents, such as anti-inflammatory drugs or targeted therapies, may offer synergistic effects in managing oxidative stress and related diseases. Research should focus on understanding how antioxidants can complement existing treatments and enhance their effectiveness while minimizing potential interactions.

While many studies have highlighted the benefits of antioxidants, there is a need for long-term research to assess their safety and efficacy. This includes evaluating potential adverse effects of high-dose antioxidant supplements and understanding their interactions with other medications or health conditions. Future research should investigate how lifestyle factors (such as diet, exercise, and smoking) and environmental exposures (like pollution and UV radiation) influence oxidative stress and the effectiveness of antioxidants. This holistic approach can provide insights into how lifestyle modifications can complement antioxidant therapies. Advancing Biomarker Discovery and Validation: Identifying and validating biomarkers of oxidative stress will improve our ability to monitor oxidative damage and evaluate the effectiveness of antioxidant interventions. Research should focus on discovering reliable biomarkers that can be used in clinical settings to assess oxidative stress levels and guide therapeutic decisions.

Exploring non-pharmacological interventions, such as dietary modifications, functional foods, and lifestyle changes, in managing oxidative stress is an important area of research. Understanding how these interventions can be integrated into daily life to enhance antioxidant defenses and prevent disease will be beneficial. As research progresses, there is a need for effective dissemination of knowledge about the role of free radicals and antioxidants in health. Public education on the benefits of a balanced diet rich in antioxidants, along with practical advice on lifestyle choices, can help individuals make informed decisions to support their health.

## Conclusion

Free radicals are highly reactive molecules with unpaired electrons that play a dual role in human health. While essential for some physiological processes, an imbalance between free radical production and the body’s ability to manage these reactive species leads to oxidative stress. This condition is implicated in various chronic diseases, including cardiovascular disorders, cancer, neurodegenerative diseases, autoimmune conditions, and diabetes mellitus.

The impact of oxidative stress is profound, causing damage to lipids, proteins, and DNA, which disrupts cellular function and contributes to disease progression. In cardiovascular diseases, oxidative stress exacerbates endothelial dysfunction and atherosclerosis. In neurodegenerative conditions, it leads to neuronal damage and cognitive decline. Cancer is often driven by oxidative DNA damage, and autoimmune diseases can result from chronic oxidative stress-induced inflammation. Diabetes mellitus complications, such as neuropathy and retinopathy, are also linked to oxidative stress.

Addressing these issues, natural compounds and antioxidants have shown promise in mitigating oxidative damage. Phenolic compounds, vitamins, and other bioactive substances derived from plants can neutralize free radicals and enhance antioxidant defenses. Flavonoids like quercetin, catechins, and anthocyanins, as well as phenolic acids such as caffeic and ferulic acids, offer significant antioxidant benefits. Vitamins C, E, and A also provide protection through various mechanisms, including direct scavenging of free radicals and modulation of cellular pathways.

Future research should focus on understanding the detailed mechanisms of free radical-induced diseases, developing novel antioxidants, and personalizing interventions. Investigating the preventive roles of antioxidants, assessing long-term safety, and exploring lifestyle impacts on oxidative stress are crucial areas for further study.
